# Continuous Monitoring of Respiratory Rate in Emergency Admissions: Evaluation of the RespiraSense™ Sensor in Acute Care Compared to the Industry Standard and Gold Standard

**DOI:** 10.3390/s18082700

**Published:** 2018-08-17

**Authors:** Christian Peter Subbe, Sean Kinsella

**Affiliations:** 1 Consultant Acute, Respiratory & Intensive Care Medicine, Bangor University & Ysbyty Gwynedd, Bangor LL57 2PW, Wales, UK; 2 PMD Solutions, Cork T12 T922, Ireland; sean.kinsella@pmd-solutions.com

**Keywords:** monitoring, respiratory rate, sensor

## Abstract

Respiratory Rate (RR) is the best marker to indicate deterioration but measurement are often inaccurate. The RespiraSense™ is a non-invasive, wireless, body worn, motion-tolerant and continuous respiratory rate monitor. We aimed to determine whether the performance of RespiraSense™ was equivalent to that of a gold standard measurement technique of capnography and the industry standard of manual counts. RespiraSense™ measures respiratory rate and transmit signals wirelessly to a tablet device. We measured respiratory rate in 24 emergency admissions to an Acute Medical Unit in the UK. Patients were observed for two hours. Manual counts were undertaken every 15 min and compared to measurements with capnography and RespiraSense™. Data from 17 patients admitted as medical emergencies was evaluated. For measurements obtained at rest a mean RR of 19.3 (SD 4.89) for manual measurements compared to mean RR of 20.2 (SD 4.54) for measurements obtained with capnography and mean RR of 19.8 (SD 4.52) with RespiraSense™. At rest, RespiraSense™ has a bias of 0.38 and limits of agreement of 1.0 to 1.8 bpm, when compared to the capnography derived RR. Measurements were within pre-defined limits of error at rest. Continuous measurement of RR with RespiraSense™ in patients admitted as acute emergencies is both feasible and reliable.

## 1. Introduction 

According to the National Institute for Health and Care Excellence [1] “respiratory rate is the best marker of a sick patient and is the first observation that will indicate a problem or deterioration in condition”. Respiratory rate is raised in respiratory conditions as well as in conditions with serious metabolic derangements such as renal failure or shock. Cuthbertson [2] found that respiratory rate had the highest area under the receiver operator characteristic curve (AUROC) for identifying patients requiring Intensive Care. Odell [3] observed that 52% of calls to a Critical Care Outreach time were for abnormalities in respiratory rate. Lam [4] stated that in patients assessed in an Emergency Department raised respiratory rate had the highest AUROC for admission to hospital. 

Accurate measurement and interpretation of this vital sign is therefore essential in ensuring best possible patient outcomes. However, despite the awareness of its importance, clinical staff find it is not efficient in a busy clinical setting [5,6,7] and have low confidence in the reliability of recordings [8]. This lack of faith is resulting in essential clinical information not being used or indeed misleading clinical care [8]. A 2015 systematic review of continuous monitoring methods found that implementation cannot yet be supported as results of clinical studies are inconclusive and the methodological quality of the studies included was questionable [9]. 

Despite its limitations manual counting of respiratory rate is seen as the industry standard whereas capnography is recognized as the gold standard measurement. We aimed to demonstrate equivalence of RespiraSense™ at measuring respiratory rate in comparison to the gold standard method of capnography [10,11] and to manual counting both when the patient is (a) instructed to remain still and (b) when allowed to go about normal hospital routines.

## 2. Materials and Methods

### 2.1. Study Design

This was a single centre, prospective, controlled, exploratory investigation designed to determine whether the performance of RespiraSense™ was equivalent to that of a gold standard measurement technique capnography and the industry standard method manual counting. The primary endpoint was the agreement between respiratory rate measured with RespiraSense™ averaged over a rolling 15-min window compared to average respiratory rate measured with capnography and by manual count. Measurements for the primary endpoint were taken for the first hour of observation only, after which the capnograph was removed. 

### 2.2. Recruitment 

Patients were recruited from the Acute Medical Unit of the Ysbyty Gwynedd, Bangor from the 8 May 2017 to the 28 June 2017.

Patients had to fulfill the following inclusion criteria: age ≥18 years, recruitment during first 24h of an acute (unplanned) admission, predicted time of at least four hours on the Acute Medical Unit. Patients were awake, self-ventilating and able to consent. Patients identified by senior clinicians for ambulatory care with an expected length of stay of less than four hours were excluded.

Patients with the following criteria were excluded: allergic to medical grade skin adhesive, pregnant women during second and third trimester, patients who were determined by the medical team to have fragile skin unsuitable for application of the adhesive of the RespiraSense™ sensor, patients under the influence of substance abuse (drug or alcohol) that may interfere with their ability to cooperate and comply with the investigation procedures, any disorder, including cognitive dysfunction, which would affect the ability to freely give full informed consent, patients whose health was judged to be deteriorating and unstable, patients with a National Early Warning Score (NEWS) [12] of >5 as a measure of deterioration and instability, patients with predominant palliative care needs.

Subjects could withdraw from the clinical investigation at any time without any rationale and without compromising their future medical care. Subjects could also be removed from a clinical investigation by their physicians if they felt it to be in the best interest of the subject. 

Once identified research participants were given the patient information leaflet and informed consent form. Measurements of manual counting of respiratory rate were shared with the clinical team to allow response to abnormal parameters and avoid duplication for the patient. 

### 2.3. Measurements 

We collected gender, age, height, weight, manually counted respiratory rate (counted over one minute in 15-min intervals), demographic data, RespiraSense™ measurements and capnography measurements. Body mass index was calculated from height and weight. 

The capnograph was performed using the LifeSense^®^ Model LS1-9R (Nonin, Plymouth, MN, USA). The monitor uses sidestream non-dispersive infrared (NDIR) spectroscopy to continuously measure respiratory rate. Calibration was performed as per manufacturer’s instructions one to two weeks before the commencement of this investigation. The Nonin LifeSense^®^ utilises a sample line (a single use disposable tubing) that attaches into the patient’s nose and connects to the monitor’s moisture trap. The cannula is inserted into each nostril and tubing placed behind the ears.

**Figure 1:**
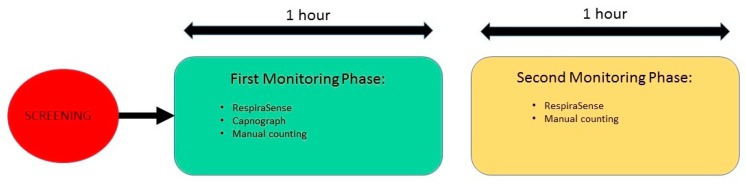
Schedule of events for this investigation.

During this investigation, the RespiraSense™ sensor was attached to the patient’s skin in the chest area using medical grade adhesive. Each subject’s participation was monitored for two hours. During the first hour patients were asked to stay supine or seated in bed to minimize movement. Capnography and manual counts were undertaken concurrently. During the second hour capnography was removed and patients were allowed to mobilize. Manual measurements continued at 15-min intervals (Figure 1). Research nurses were directly observing patients and filed diaries with activities. 

### 2.4. RespiraSense

**Figure 2:**
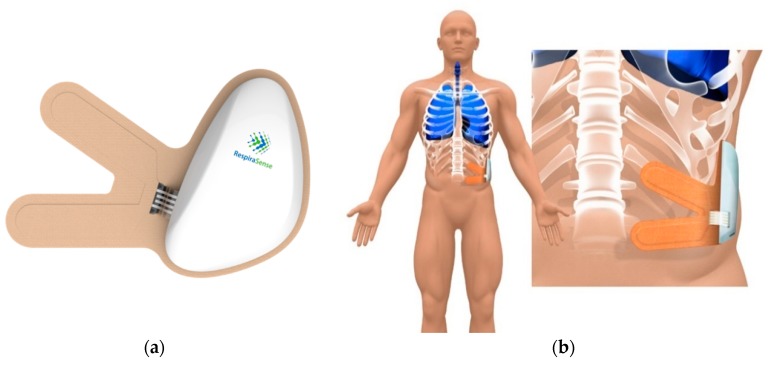
RespiraSense™ lobe and sensor (**a**) and position while undertaking monitoring (**b**).

RespiraSense™ is a CE-approved device (Figure 2) and as such has been deemed safe to use with no expected interactions with most concomitant medical treatments [13]. As per the Instructions For Use (IFU) RespiraSense™ is not to be used during defibrillation, Magnetic Resonance Imaging, X-ray or other medical imaging procedures.

RespiraSense™ is a non-invasive, wireless, body worn, motion-tolerant and continuous respiratory rate monitor [14]. The system consists of a RespiraSense™ Lobe, a single-use Sensor (Figure 2a) and a Mobile Application. The Lobe and Sensor—‘RespiraSense™’—are assembled together and placed onto the patient for the purpose of monitoring respiratory rate. RespiraSense™ utilises a generic sensory technology called piezoelectric films which are assembled into an array. This piezoelectric array is able to measure small deformations in the relative angles of the thoracic and abdominal surfaces that occur during breathing and convert these changes into an electrical signal. The rate of these changes corresponds to respiratory rate. On-board accelerometers and discriminatory algorithms in the device means that the system is tolerant to movement artefacts such as coughing, talking and walking.

Respiratory rate data collected by RespiraSense™ are transmitted to the mobile application via Bluetooth. This data can then be transmitted via Wi-Fi to the most appropriate location in the hospital, be that a nurse’s station or the Electronic Health Record (EHR). Access to the mobile stored data was only available to the research team. Respiratory rates were shared with the clinical teams looking after the study patients.

### 2.5. Statistics 

The primary endpoint was analysed using a Bland Altman (BA) analysis to measure limits of agreement between methods. The BA analysis was corrected for repeated measures within each subject if required. In addition, a Deming regression was performed on the collected data. The pass criteria for the study were defined as: The limits of agreement of the Bland Altman analysis to not contain +3 bpm or −3 bpm, with the 95% CI on the Deming regression intercept containing 0 and the 95% CI on the Deming regression slope containing 1. 

The sample size was calculated using previously collected unpublished data of RespiraSense™ versus capnograph. The expected standard deviation in difference in average RR over a 15-min window between RS and capnography was estimated to be 1.1 bpm. A 2-sided 95% CI for the mean of the differences between methods were constructed with a maximum length of 2 bpm. Assuming a dropout rate of 10% and at least 1 usable data point per patient, this required the recruitment of 21 patients. The study had thus 90% power to detect a difference of less than 5% between respiratory rate as measured by the capnograph and respiratory rate measured by RespiraSense™. 

All data were summarised with descriptive statistics including mean, standard deviation (SD), median, minimum, maximum, first and third quartiles for interval scales and number and frequency for categorical variables.

### 2.6. Ethics 

RespiraSense™ is a CE-labelled product that was being used within its intended purpose. The investigational study was performed in accordance with the ethical requirements defined in the Declaration of Helsinki and to the principles set out in the ISO 14155 international standard and with national regulations. The study was approved by the research ethics committee on the 17 March 2017 (REC reference 17/WA/0083). 

## 3. Results 

### 3.1. Recruitment 

**Figure 3:**
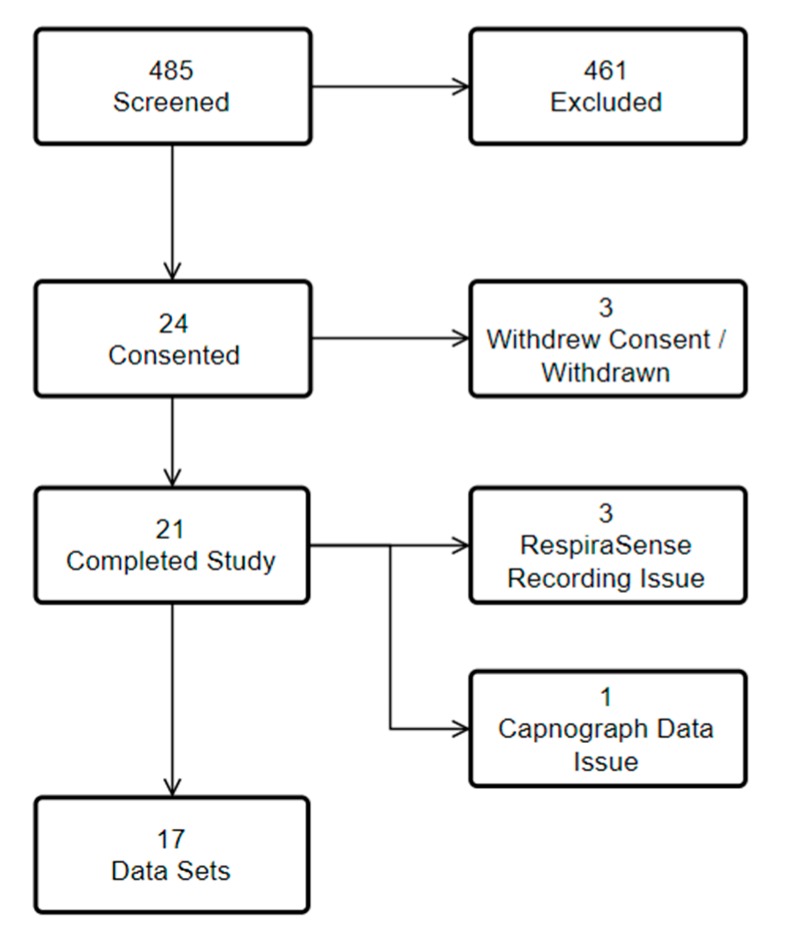
Recruitment into study with exclusions.

Data were collected from 8 May 2017 to 28 June 2017. 24 patients met inclusion criteria, of these 24 agreed to consent. In all, 21 patient’s measurements were completed (Figure 3). Of the 461 patients who were excluded 98 were not suitable/ineligible, 78 were unable to consent, 65 were discharged home, 60 were awaiting further investigations, 50 could not be included as the research nurse was seeing another patient, 27 were on oxygen, 22 were transferred to another ward/hospital, 19 had fragile skin, in 17 no resource was available, 16 declined, five were asleep, one was known to the research nurse, for one no reason was given, and two had errors with documentation or equipment.

Data from 17 patients were included in the analysis. 11 (65%) patients were male and the mean age of patients was 62 years (SD 22). One patient was unable to stand for measurements and was excluded from BMI reporting. Median Body Mass Index was 26.6 (IQR 6.3), 15 patients had a BMI of over 20, nine patients had a BMI of over 25. 

Sixty two data points were available for analysis of the primary endpoint. For manual counting, at rest, 64 data points were available for analysis and for manual counting, in motion, 79 data points were available for analysis.

### 3.2. Respiratory Rate Measurements at Rest

Manual measurements were taken in 15-min intervals. There were matched to measurements of respiratory rate obtained with capnography and RespiraSense™. 

**Figure 4:**
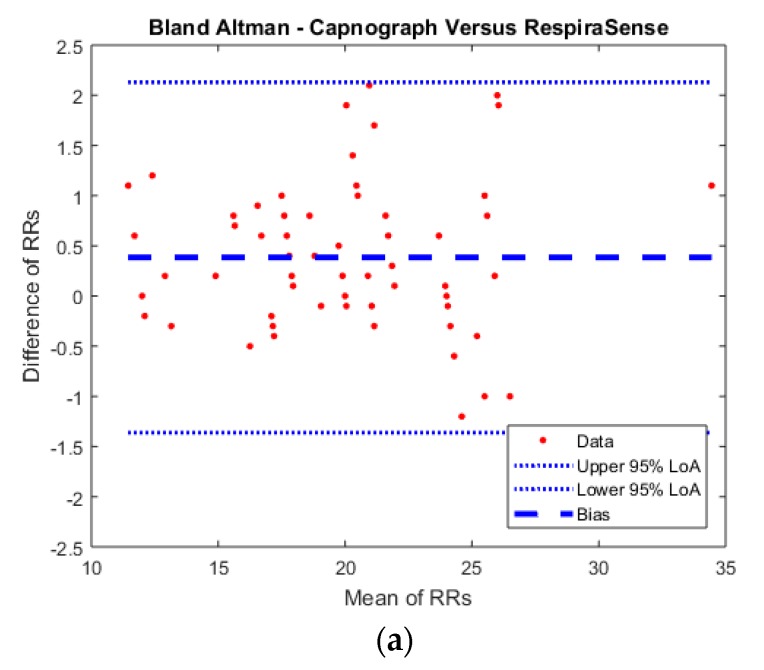

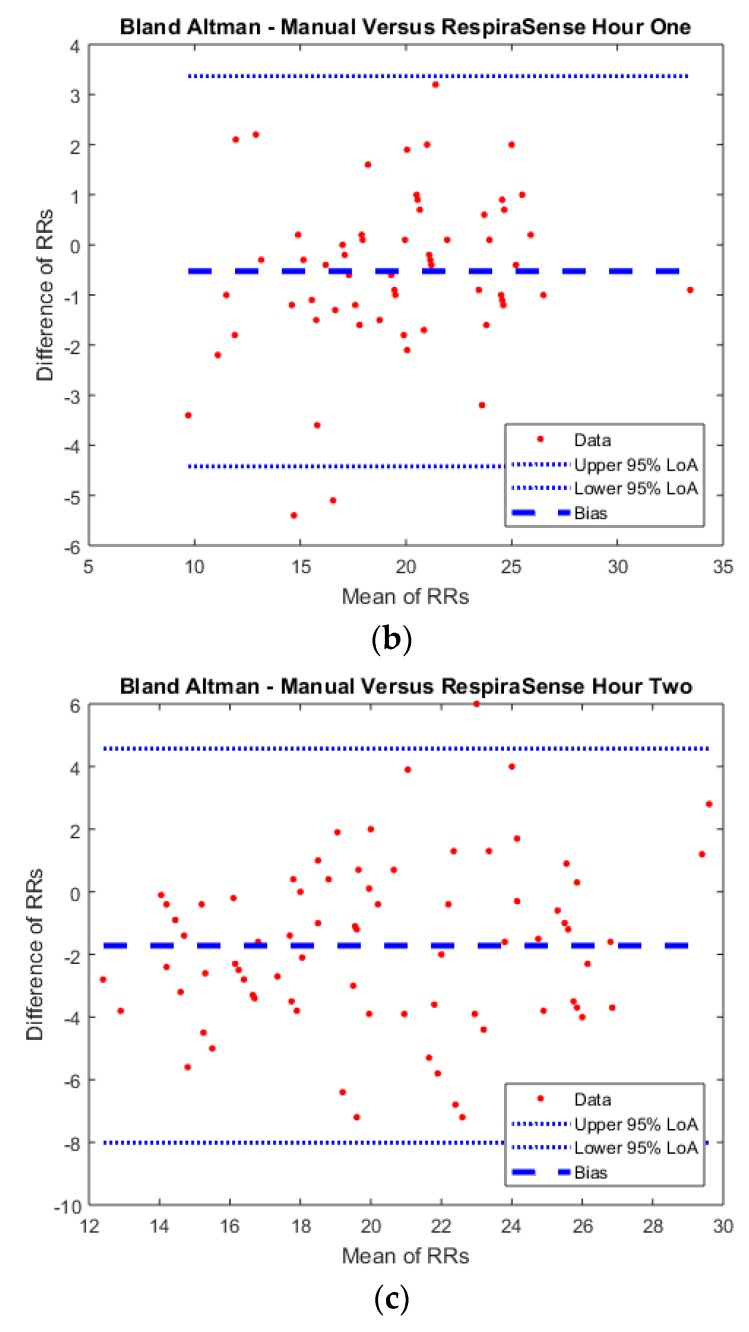
Bland Altman plots of (**a**) Capnograph counts vs. RespiraSense in the first hour (at rest); (**b**) Manual respiratory rate counts vs. RespiraSense in the first hour (at rest); (**c**) Manual respiratory rate counts vs. RespiraSense in the second hour (while moving).

For measurements obtained at rest a mean RR of 19.3 (SD 4.89) for manual measurements compared to mean RR of 20.2 (SD 4.54) for measurements obtained with capnography and mean RR of 19.8 (SD 4.52) with RespiraSense™. The range of measurements for individual patients obtained during the full hour varied by a mean RR of 3.9 (SD 2.17) for manual measurements compared to mean RR of 2.4 (SD 2.37) for measurements obtained with capnography and mean RR of 2.3 (SD 2.27) with RespiraSense™. The results of the measurements are presented in the BA plots below (Figure 4). Bland Altman analysis was corrected for repeated measures within subjects. 

At rest, RespiraSense™ had a bias of 0.38 and limits of agreement of 1.0 to 1.8 bpm, when compared to the capnography derived RR (R^2^ = 0.99). RespiraSense™ had a bias of −0.70 and limits of agreement of −4.9 to 3.5 bpm, when compared to the manually counted RR (R^2^ = 0.90). Agreement of measurements was within pre-defined limits for capnograph versus RespiraSense™. 

There was a difference (p = 0.0062) in respiratory rate measured with RespiraSense™ in men (mean 21.4 bpm) and women (mean 19.5 bpm). There was a difference (p < 0.0001) in respiratory rate measured with RespiraSense™ in patients with BMI lower than 25 (mean 22.3 bpm) and those with BMI greater than or equal to 25 (mean 18.1 bpm). There was a difference (p = 0.0002) in respiratory rate measured with RespiraSense™ of less than 70 years of age (mean 21.6 bpm) and those of 70 years of age or greater (mean 19.4 bpm).

### 3.3. Respiratory Rate Measurements during Period with Permission of Movement

Manual measurements were taken in 15-min intervals. There were matched to measurements of respiratory rate obtained RespiraSense™. For measurements obtained at rest a mean RR of 19.34 (SD 4.61) for manual measurements compared to a mean RR of 21.1 (SD 4.15) with RespiraSense™. The range of measurements obtained during the full hour varied by a mean RR of 4.3 (SD 2.65) for manual measurements compared to mean RR of 3.2 (SD 1.55) for measurements obtained with RespiraSense™. With movement RespiraSense™ had a bias of −1.72 and limits of agreement of −6.8 to 3.3 bpm, when compared to the manually counted RR (R^2^ = 0.83). The results of the measurements are presented in the BA plots below (Figure 4). 

When comparing RR measurements during the hour of resting with RR measurements obtained in the second hour, the mean RR decreased by 0.06 breath (SD decreased by 0.28) for manual measurements. For measurements taken at the same time with RespiraSense™, the mean RR decreased by 1.26 breaths per minute (SD decreased by 0.37). 

### 3.4. Identification of Periods of Movement from Movement Sensor

**Figure 5:**
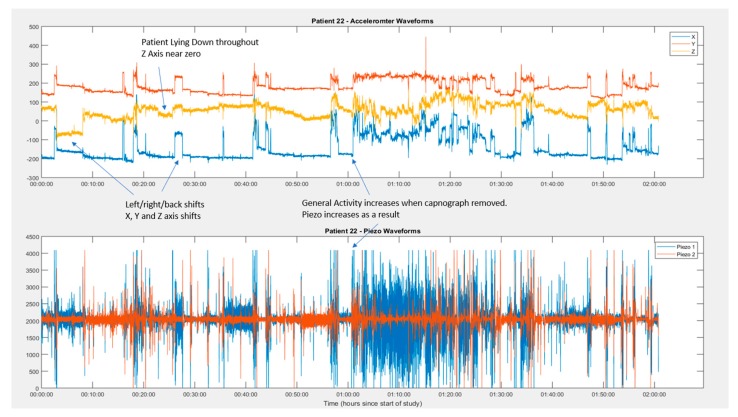
Example of detection of movement artefacts.

This movement detection is performed as an automatic part of the RespiraSense™ algorithm Movement was identifiable from movement sensor readings through <1% of the study period (Figure 5). Though the automatically detected movement percentage was low, the measure of patient activity was 45% higher in the second hour than the first. 

## 4. Discussion 

In the present study we have shown that RespiraSense™ delivers a high quality measurement of respiratory rate compared to gold standard and industry standard in patients admitted as emergencies to an Acute Medical Unit. Respiratory rate was measured within the pre-defined level of agreement at rest. BMI, gender and age of patients were recorded in the study and did not have an impact on the ability to obtain reliable results. Period of movement could be identified from tracings of the movement sensor of the device (Figure 5), this is important given that currently used normal ranges for physiological parameters assume a measurement at rest. 

Respiratory rate is a key measurement for risk stratification of cardiac and respiratory conditions and trauma [15]. Despite this it has been called the ‘neglected’ vital sign [15]. Documented measurements often diverge from accurately counted ones [7]. While there are respiratory sensors in development using a range of methods very little data is available on usage outside of controlled laboratory settings [16,17,18,19,20].

This was a prospective study powered to show the reliability of respiratory rate measurements in a ‘real life’ clinical environment. The fact that the study was undertaken in an Acute Medical Unit with patients admitted as emergencies with often raised respiratory rate adds face validity to the usability of the device in usual clinical practice. We made a conscientious decision not to include highly unstable emergencies given the ethics concerns of consenting patients in this patient group and the more limited access in patients undergoing active resuscitation. There is hence still missing evidence in this particular patient group. 

While the present study is a single centre study we believe that physiology is sufficiently universal to allow extrapolation of the results to other hospitals or countries. The new technology will help to get more reliable recording of respiratory rate with more precise assessment of physiology and the risk contained in the measurement of respiratory rate but it is noteworthy that respiratory rate and variability might increase with movement. Our sample was too small to derive conclusions and further research in this area is required. The choice of the ‘right respiratory rate’ for risk stratification will therefore require further thoughts. Given that the movement sensor was able to reliably identify movement we believe that it might be possible to restrict measurements of respiratory rate to periods of rest only. The duration of battery life make the unit potentially suitable for short admissions to hospital, intensive monitoring to elicit improvement or deterioration in patients in whom an admission could be averted after a short period of assessment, periods of acute deterioration on general wards or monitoring in step-down units with limited length of stay. 

More work is needed to determine the clinical significance of measurements of respiratory rate during movement. Patients with a steeper or larger rise for a given level of movement/exertion might be sicker or less fit than those with a small increase under comparable circumstances. Clinical trials need to quantify the number of patients identified in whom clinical decisions could be usefully supported with the additional high quality data that the RespiraSense™ sensor is able to provide. 

## 5. Conclusions

Continuous monitoring of respiratory rate is possible even in patients who are acutely unwell and mobile in an acute care setting. This opens up a possibly more reliable route to identify catastrophic deterioration in this patient group. The impact on monitoring protocols and existing algorithms requires further investigation.
